# NLRSeek: A reannotation–based pipeline for mining missing NLR genes in sequenced genomes

**DOI:** 10.1016/j.abiote.2025.100001

**Published:** 2025-10-17

**Authors:** Mengda Wang, Xiaowei Fu, Li Yin, Yi Zhao, Qingjie Yang, Xuteng Ye, Jun Cheng, Daolong Dou, Jinding Liu, Gan Ai

**Affiliations:** College of Plant Protection, Academy for Advanced Interdisciplinary Studies, Nanjing Agricultural University, Nanjing, 211800, China

**Keywords:** NLRs (nucleotide-binding leucine-rich repeat receptors), R genes, Disease resistance breeding, Bioinformatics

## Abstract

Nucleotide-binding leucine-rich repeat (NLR) proteins function as receptors and signaling factors in plant immune systems. Identifying the genes encoding NLR proteins in genomic sequences provides crucial information for breeding disease-resistant crops. However, NLR proteins are frequently misannotated during automated proteome prediction and downstream identification tools that rely on proteomic data struggle to recover these missing NLRs. To address this problem, we developed NLRSeek (https://github.com/Wang-Mengda/NLRSeek), a genome reannotation–based pipeline for NLR identification. This workflow integrates *de novo* detection of NLR loci at the genome level with targeted genome reannotation, systematically reconciling these results with existing annotations to produce a comprehensive set of NLR predictions. Our pipeline identified a larger number of NLRs than other NLR annotation tools: even in the well-annotated model plant *Arabidopsis thaliana*, NLRSeek identified a previously unannotated NLR gene whose expression and translation were confirmed by transcriptome and ribosome-profiling data. The NLRSeek pipeline showed particularly strong performance for non-model species with incomplete annotations. For example, in the yam species *Dioscorea zingiberensis*, *Dioscorea tokoro*, and *Dioscorea dumetorum*, NLRSeek identified 33.8 ​%–127.5 ​% more NLR genes than conventional methods. Importantly, 45.1 ​% of the newly annotated NLRs exhibited detectable expression, suggesting that they are true genes that were previously overlooked. Analysis of the newly identified sequences revealed that NLRs have undergone expansion in *D. zingiberensis* through tandem duplication, an insight that was not attainable using previous NLR annotation tools. Our novel NLR identification pipeline may reveal untapped genetic resources for engineering disease-resistant crops.

## 1 Introduction

Plants have evolved a sophisticated innate immune system to prevent infection by diverse pathogens [1]. Successfully adapted pathogens deliver effectors to suppress host defense responses and thus facilitate infection [2,3]. As a countermeasure, plants have evolved specific intracellular receptors, the nucleotide-binding leucine-rich repeat (NLR) proteins, that recognize pathogen effectors and activate effector-triggered immunity (ETI) [4]. ETI provides robust defense responses that typically include the hypersensitive response, a type of localized cell death at the infection site that inhibits pathogen spread [5,6].

NLRs are divided into three classes based on their N-terminal domains: the Toll/interleukin-1 receptor/R (TIR)-NLRs, the coiled-coil (CC)-NLRs, and the resistance to powdery mildew 8 (RPW8)-NLRs (TNLs, CNLs, and RNLs, respectively) [7,8]. Outside of the N-terminal domain, most NLRs share the same general structure, consisting of a central NB-ARC domain (a nucleotide-binding domain shared by apoptotic protease-activating factor-1 [Apaf-1], resistance [R] proteins, and *Caenorhabditis elegans* death-4 protein [CED-4]) and a C-terminal LRR domain [9]. In general, the N-terminal domain mediates downstream immune signal transduction; the NB-ARC domain binds ATP/ADP and has AAA-ATPase activity, functioning as a molecular switch to link immune sensing to signal transduction; and the LRR domain mediates recognition specificity through protein–protein interactions [10,11].

Because NLRs act as receptors for pathogen-specific factors, the identification of NLRs is an important step in breeding crops for disease resistance. However, NLR identification remains challenging for two main reasons. First, NLRs have complex genomic architectures characterized by multiple exons, long introns, and frequent rearrangements and duplications [12]. Second, they are typically expressed at low levels. Both of these issues hinder accurate and comprehensive annotation of NLRs through automated pipelines [13]. Current NLR prediction tools are either genome-based (e.g., NLR-Annotator [14]) or proteome-based (e.g., NLRtracker [15]). Genome-based tools excel in detecting genomic NLR loci but lack structural resolution, whereas proteome-based tools exhibit high sensitivity for NLR detection but require high-quality annotation.

Here, we present NLRSeek, an NLR identification pipeline based on genome reannotation. This workflow identifies NLR loci at the genomic level and performs local genome re-annotations to provide a comprehensive set of NLR predictions. Compared with conventional methods, NLRSeek identified a larger number of NLR genes in both model and non-model species, including a newly annotated Arabidopsis (*Arabidopsis thaliana*) NLR whose expression and translation were confirmed by transcriptome and ribosome-profiling data. The NLRSeek pipeline thus recovers NLRs that are missing from most automated genome annotations, enabling the systematic exploration of plant immune repertoires to accelerate resistance breeding.

## 2 Results

### 2.1 Genome reannotation–based NLR prediction

**Fig. 1:**
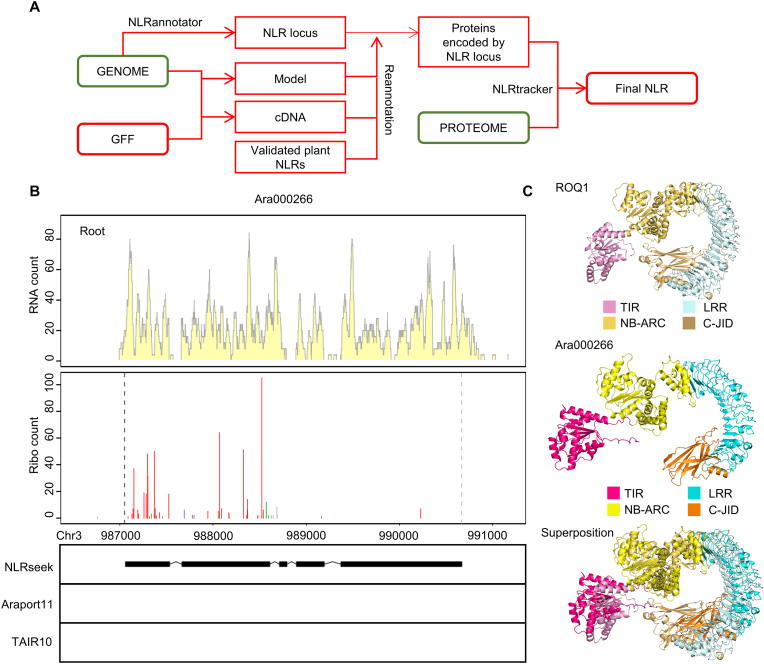
Genome reannotation–based NLR prediction. **A** The NLRSeek pipeline for NLR prediction. NLR-Annotator (v2.1) is used to identify putative NLR loci across the genome, followed by reannotation. The protein sequences encoded by newly annotated genes and the existing proteome are analyzed with NLRtracker to predict NLRs. Redundant newly annotated NLR sequences that overlap with the existing proteome are removed. **B** Expression and translation evidence for Ara000266. The genome annotations of the Ara000266 locus from NLRSeek, Araport11, and TAIR10 are shown in the lower panel. RNA-seq and Ribo-seq data from *Arabidopsis* roots were mapped to the genome. The upper panel displays genome browser views of the RNA-seq and Ribo-seq signals; the y-axes indicate RNA-seq counts and Ribo-seq P-site counts. **C** Structural comparison between the protein encoded by Ara000266 and ROQ1. The AlphaFold3-predicted structure of the protein encoded by Ara000266, the reported ROQ1 structure (PDB: 7JLU), and their superimposition are shown.

We developed NLRSeek, a genome reannotation–based pipeline for the identification of NLRs that have previously escaped automated annotation (Fig. 1A). The pipeline consists of three stages. First, we perform *de novo* searches for NLR loci in the genome using NLR-Annotator [14], a highly precise tool for NLR locus identification that operates independently of existing annotations. Next, we re-annotate the NLR loci using a tool that integrates trained gene models with experimentally validated plant NLR sequences, enabling accurate structural predictions of previously overlooked genes [16]. Finally, we merge the newly annotated protein sequences with the original proteome annotations and process them through NLRtracker [15]—a high-precision proteome-based classifier—for definitive NLR identification and classification. This integrated approach combines genomic and proteomic evidence to recover NLR genes that are absent from the original genome annotation. We have encapsulated the entire workflow in a user-friendly software package that is available for download from our GitHub repository (https://github.com/Wang-Mengda/NLRSeek), facilitating its use in plant immunity research and resistance breeding programs.

To evaluate the performance of the NLRSeek pipeline, we used it to predict NLR genes in the *Arabidopsis* genome and compared the results with those of NLRtracker [15], NLR-Annotator [14], Resistify [17], and RRGPredictor [18]. NLRSeek identified 173 NLR genes in *Arabidopsis* (Table S1), more than were identified by NLR-Annotator (164), NLRtracker (172), Resistify (167), and RRGPredictor (158) (Fig. S1). Although NLRtracker can exhibit reduced accuracy in predicting the highly variable CC domains frequently missed by InterProScan [17]**,** it remains one of the most sensitive and accurate NLR prediction pipelines. Nonetheless, our NLRSeek pipeline identified an additional NLR gene (designated as Ara000266) that was missed by NLRtracker (Fig. 1B)**.** This NLR is a typical member of the TNL class, with complete start and stop codons, but it was annotated as a pseudogene in the original automated annotation version Araport11 or TAIR10 (Fig. 1B).

To test whether Ara000266 is truly an expressed gene, we analyzed RNA-sequencing (RNA-seq) and ribosome profiling (Ribo-seq) data [19], which confirmed that it was expressed and translated in roots (Fig. 1B) and shoots (Fig. S2A). Three-dimensional structure modeling and alignment revealed that the protein encoded by Ara000266 contains complete TNL domains highly similar to those of previously published TNLs (Fig. 1C), including a C-terminal jelly-roll/Ig-like domain (C-JID), which has been reported to participate in the recognition of pathogen avirulence proteins [20]. Additional analysis identified a conserved catalytic site for NAD+ ​hydrolysis in the TIR domain (Fig. S2B), providing further evidence that Ara000266 is a functional gene rather than a pseudogene. These results confirm the accuracy of the NLRSeek prediction and demonstrate that misannotation of NLRs can occur even in well-studied model plants like *Arabidopsis*.

We next expanded our comparative analysis to the wheat (*Triticum aestivum*), rice (*Oryza sativa*), and banana (*Musa acuminata*) genomes (Fig. S1), in which NLRSeek identified 471, 1845, and 367 NLR genes, respectively (Table S2–4). NLRSeek identified more NLR genes than other annotation methods for all three species (Fig. S1), and >96 ​% of the NLRs identified by other methods were also detected by NLRSeek (Fig. S3).

### 2.2 NLR prediction in non-model Dioscorea species

**Fig. 2:**
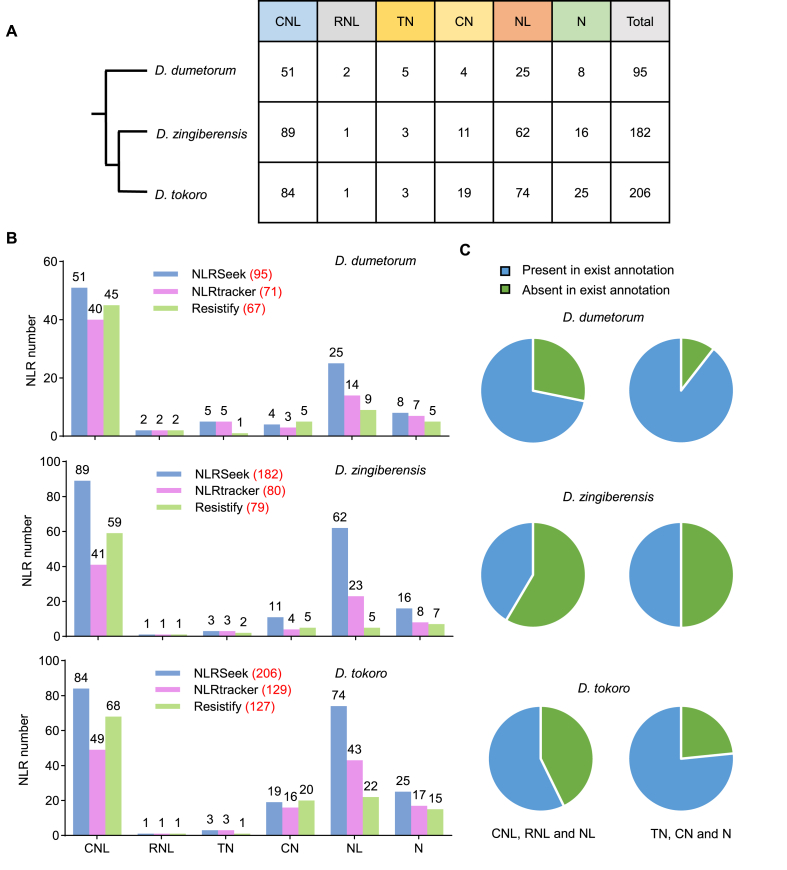
Comparison of NLRSeek with existing tools for NLR identification in three *Dioscorea* species. **A** Phylogenetic tree illustrating the numbers of NLRs identified in three *Dioscorea* species. CNL: CC-NB-ARC-LRR, RNL: RPW8-NB-ARC-LRR, TN: TIR-NB-ARC, CN: CC-NB-ARC, NL: NB-ARC-LRR, N: NB-ARC. **B** Numbers of NLRs identified by NLRSeek, NLRtracker, and Resistify in three *Dioscorea* species. **C** NLRs that contain LRR domains are more frequently missed by annotation pipelines.

Yam (*Dioscorea* spp.) is widely cultivated as an essential crop in tropical and subtropical regions of Southeast Asia, Africa, and Latin America [21]. The identification of NLR genes in yam for resistance breeding is a key focus of our laboratory's research. As non-model plants, yam species do not yet have well-annotated genomes, presenting an ideal opportunity to test the efficacy of our newly developed pipeline. NLRSeek predicted 182 NLR genes in *Dioscorea zingiberensis*, 206 in *D. tokoro*, and 95 in *D. dumetorum* (Fig. 2A, Table S5–7). Compared with NLRtracker, our pipeline identified 24 additional NLRs (33.8 ​% more) in *D. dumetorum*, 102 additional NLRs (127.5 ​% more) in *D. zingiberensis*, and 77 additional NLRs (59.7 ​% more) in *D. tokoro* (Fig. 2B). Similar results were obtained when NLRSeek was compared with Resistify (Fig. 2B). Consistent with previous research suggesting that TNL genes were lost in the common ancestor of monocots [7], no TNLs were identified in these yam species. However, several proteins with only a TIR domain and an NB-ARC domain (termed TNs) were identified (Fig. 2B).

Between 11 ​% and 50 ​% of the TN-, CN-, and N-type NLRs identified by NLRSeek were absent from the original genome annotations, compared with 28 ​%–57 ​% of the RNLs, CNLs, and NL-type NLRs (Fig. 2C). This pattern suggests that annotation pipelines may be more likely to overlook NLRs that contain LRR domains, consistent with previous reports that LRR-containing sequences can be mistaken for repetitive elements and filtered out during automated genome annotation [12].

### 2.3 Newly identified NLR genes in D. zingiberensis are transcribed

**Fig. 3:**
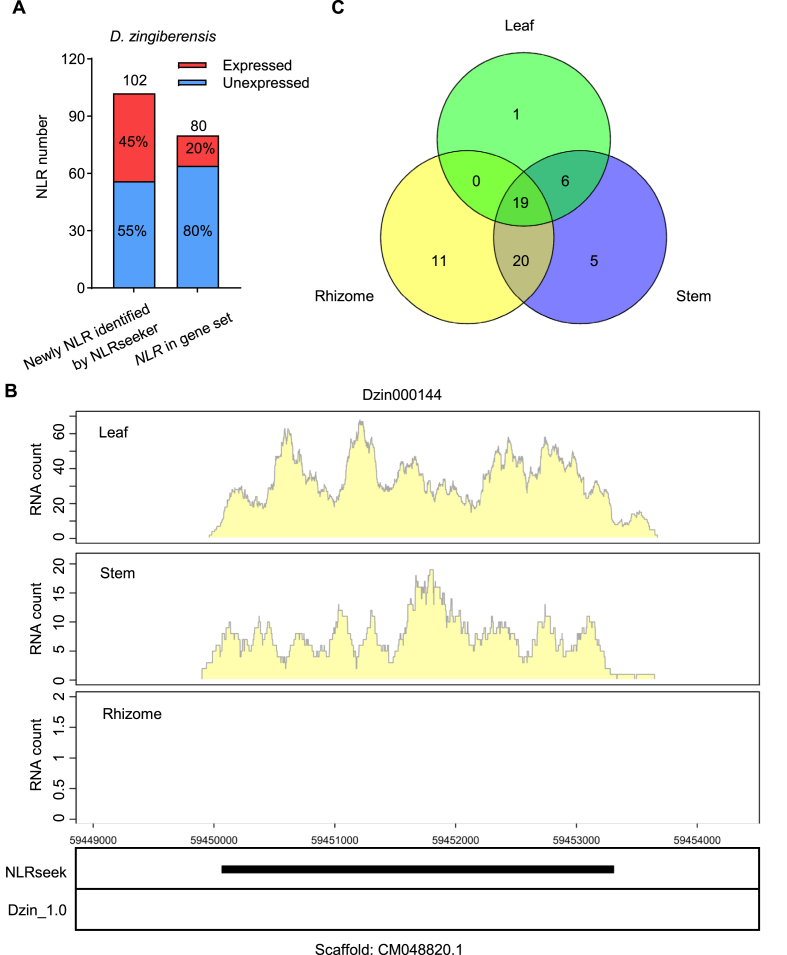
The newly annotated NLR genes in *D. zingiberensis* are transcribed. **A** Numbers of NLRs and percentages of expressed NLRs in three *Dioscorea* species. **B** Expression evidence for Dzin000144. The lower panel shows annotations of the Dzin000144 locus from NLRSeek and from the original genome annotation (Dzin_1.0). The upper panels show genome browser views of RNA-seq signals from *D. zingiberensis* stem, leaf, and rhizome tissues. The y-axes indicate RNA-seq read counts. **C** Numbers of *D. zingiberensis* NLRs expressed in different tissues.

To further assess the reliability of the NLRSeek predictions, we used *D. zingiberensis* as a test case to examine whether the newly annotated NLR genes are truly transcribed. RNA-seq data from rhizome, stem, and leaf tissues revealed that 62 NLRs were expressed in at least one tissue (FPKM >1) (Table S5). Of the 102 newly annotated NLRs, 46 (45.1 ​%) are expressed, while 16 of the 80 NLRs from the original genome annotation (20 ​%) were expressed (Fig. 3A–S4). For example, Dzin000144 was a newly annotated CNL that was clearly expressed in stems and leaves but not in rhizomes (Fig. 3B). This proportion of expressed genes strongly suggests that the previously unannotated NLRs are in fact functional genes rather than annotation artifacts.

Seventeen NLRs were expressed in only one tissue, 26 in two tissues, and 19 in all three tissues examined (Fig. 3C–S4). This pattern indicates a degree of tissue specificity in NLR gene expression, perhaps reflecting the roles of individual NLRs in combating different pathogens. These results also demonstrate that a significant number of NLR genes are likely to be overlooked if genome annotation relies solely on transcriptome data from a small number of tissue types.

### 2.4 NLR genes form clusters in the D. zingiberensis genome

Most of the *D. zingiberensis* NLR genes (167 of 182) were mapped to chromosomes, but fifteen were located on scaffolds. Their chromosomal distribution was uneven, with ∼50 ​% of the *NLRs* located on chromosomes 3 and 4 (CM048820.1 and CM048821.1) (Fig. S5A and B), and there was no significant correlation between chromosome length and the number of NLR genes per chromosome.

Multiple NLR genes within an interval of less than 250 ​kb were considered to form a cluster [[22], [23], [24]], and the *D. zingiberensis* NLR genes were thus grouped into 34 clusters (142 NLR genes) and 40 singletons (Fig. S5A). NLR gene clusters were present on the majority of chromosomes, including chromosomes 1, 2, 3, 4, 5, 8, and 9, but were absent on chromosomes 6, 7, and 10, likely owing to the low numbers of NLR genes on these chromosomes. The largest cluster, located on chromosome 4, contained 15 NLR genes, whereas the smallest clusters contained only two genes. These data indicate that NLR genes form clusters in the *D. zingiberensis* genome, an observation made possible by the prediction of new NLR genes using NLRSeek (Fig. S5A).

### 2.5 Tandem duplication has driven the expansion of NLR genes in D. zingiberensis

**Fig. 4:**
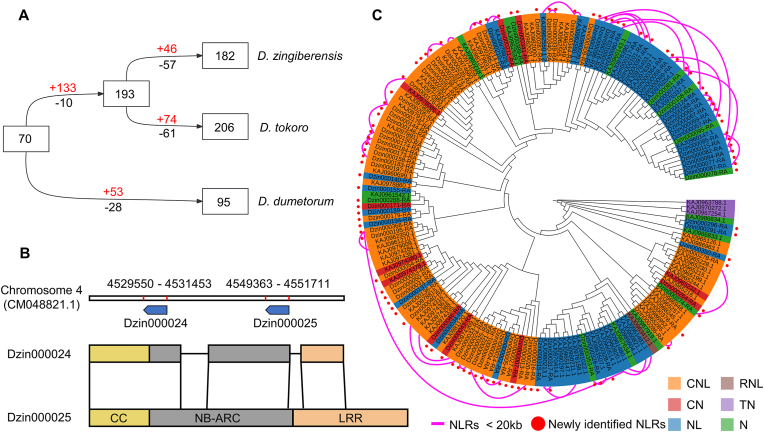
Tandem duplication has driven the evolutionary expansion of NLRs in *D. zingiberensis*. **A** Estimation of NLR gene numbers in ancestral species and their gains/losses in extant *Dioscorea* species. Numbers in rectangles are gene numbers in ancestral and extant species. Plus and minus signs indicate the numbers of genes gained and lost, respectively. **B** Example of tandem duplication. The upper panel shows the genomic locations of Dzin00024 and Dzin00025, and the lower panel displays their gene structures. Homologous regions are connected by black lines. **C** Phylogenetic tree of *D. zingiberensis* NLRs. NB-ARC domains were used to construct the tree. NLRs newly identified by NLRSeek are marked with red dots. Purple lines connect NLR pairs located within 20 ​kb of each other in the genome.

We next used the newly predicted NLR genes to examine the evolutionary history of this gene family in *Dioscorea*. We estimated the number of NLRs in the most recent common ancestor (MRCA) of *D. zingiberensis, D. dumetorum*, and *D. tokoro*, as well as the numbers of NLRs gained and lost during the evolution of each species. We used the modified reconciled-tree method, which is based on comparing a bootstrap condensed gene tree with the species tree [25]. As shown in Fig. 4A, there were 70 NLRs in the MRCA, 193 NLRs in the common ancestor of *D. zingiberensis* and *D. tokoro* (133 NLR genes gained and 10 lost) and 95 NLRs in the *D. dumetorum* lineage. After divergence from *D. tokoro*, the *D. zingiberensis* lineage experienced 46 gain and 57 loss events, resulting in a final repertoire of 182 NLR genes.

Phylogenetic analysis based on the NB-ARC domains of NLRs from the three yam species revealed that *D. zingiberensis* NLRs tended to cluster together in the phylogenetic tree (Fig. S6), indicating species-specific NLR expansions in *D. zingiberensis*. Indeed, we observed that 41 of the *D. zingiberensis* NLR genes were located <20 ​kb apart in the genome, suggesting that tandem duplication events had driven the evolutionary expansion of these NLRs. For example, Dzin000024 showed high similarity with Dzin000025, and both were located within 20 ​kb on chromosome 4 (Fig. 4B). Similarly, the phylogenetic analysis revealed high sequence similarity among NLRs located within 20 ​kb of each other (Fig. 4C). If the newly annotated NLRs were excluded, only four of the originally annotated NLRs exhibited this phenomenon, obscuring the true pattern of tandem duplications (Fig. 4C). These results highlight the exceptional utility of NLRSeek for evolutionary analysis of NLRs.

## 3 Discussion

We developed a genome reannotation pipeline for the detection of missing NLRs in plant genomes. NLRSeek identified larger numbers of NLRs than other annotation methods in both model and non-model plants, and many of the newly identified NLRs were clearly expressed, indicating that they were not false positives. The identification of new NLRs in *D. zingiberensis* enabled us to demonstrate that tandem duplication has driven expansion of the *NLR* gene family in this species.

The additional NLRs detected in three yam species (127.5 ​%, 59.7 ​%, and 33.8 ​% more than were present in the original annotations of *D. zingiberensis*, *D. tokoro*, and *D. dumetorum*, respectively) highlight a substantial gap in our understanding of the immune receptor repertoires of these economically important crops. The discovery of NLRs misannotated as pseudogenes or completely missed by conventional annotation methods underscores the need for specialized tools dedicated to NLR identification. Our data also suggest that NLRs with LRR domains are more likely to be overlooked during automated annotation (Fig. 2B), perhaps owing to their repetitive structures [12].

In conclusion, this study introduces an improved methodology for NLR identification and reveals fundamental insights into the evolutionary dynamics of NLRs in *D. zingiberensis* and related species. NLRSeek can help researchers identify and utilize the full complement of NLR genes in their species of interest, providing new targets for use in crop resistance breeding.

## 4 Materials and methods

### 4.1 Genome data used in this study

Genome assemblies, gene annotation files, and protein sequences were obtained from the following sources: *A. thaliana* from EnsemblPlants (TAIR10.59; https://plants.ensembl.org/Arabidopsis_thaliana/Info/Index), *D. zingiberensis* from the National Center for Biotechnology Information (NCBI) (GCA_026586065.1; https://www.ncbi.nlm.nih.gov/datasets/genome/GCA_026586065.1/), *D. dumetorum* from the University of Bielefeld publication repository (https://pub.uni-bielefeld.de/record/2941469), *D. tokoro* from the Iwate Biotechnology Research Center (https://genome-e.ibrc.or.jp/resource/dioscorea-tokoro/), *O. sativa* from NCBI (GCF_034140825.1; https://www.ncbi.nlm.nih.gov/datasets/genome/GCF_034140825.1/), *T. aestivum* from NCBI (GCF_018294505.1; https://www.ncbi.nlm.nih.gov/datasets/genome/GCF_018294505.1/), and *M. acuminata* from NCBI (GCF_036884655.1; https://www.ncbi.nlm.nih.gov/datasets/genome/GCF_036884655.1/).

### 4.2 Genome reannotation–based NLR prediction pipeline

First, putative NLR loci were identified across the genome using NLR-Annotator (v2.1). For each locus, the 4000-bp upstream and downstream genomic sequences were extracted. Species-specific gene-structure prediction models were trained using AUGUSTUS [26] and SNAP [27] based on the corresponding genome assemblies and gene annotation files. Experimentally validated plant NLR sequences were retrieved from PRGdb 4.0 (http://prgdb.org/prgdb4/) [28] and RefPlantNLR (https://doi.org/10.1371/journal.pbio.3001124.s013) and used as homologous reference proteins. MAKER software [29] (v2.31.11) was used to re-annotate the extracted genomic fragments by integrating the trained gene models, the experimentally validated plant NLR sequences, and the target species’ cDNA sequences. The protein sequences encoded by the newly annotated genes and the NLRs from the original proteome were then used as input for NLRTracker tools to predict NLRs. Redundant newly annotated NLR sequences that overlapped with the existing proteome were removed (Fig. 1A).

### 4.3 Transcriptome analysis

RNA-seq and Ribo-seq data for *Arabidopsis* were obtained from the NCBI Gene Expression Omnibus (GSE81332) [19]. Raw sequencing data were quality-filtered using the FASTX-Toolkit (v0.0.14) [30] with parameters -q 20 -p 85 to remove low-quality reads. Adapter sequences (AGATCGGAAGAGCACACGTCT) were trimmed using FASTX-clipper. For Ribo-seq data, only adapter-clipped reads were retained for downstream analyses. The trimmed Ribo-seq reads were aligned to known *Arabidopsis* contaminant RNAs, including rRNAs, tRNAs, and snoRNAs, using Bowtie2 [31] with the -L 20 parameter. Unaligned reads were then mapped to the *Arabidopsis* reference genome (TAIR10.59) using STAR [32]. P-site positions from the Ribo-seq data were determined using RiboTaper [33]. RNA-seq and Ribo-seq read distributions were visualized using RiboplotR [34]. For RNA-seq data, quality control was performed using fastp [35], and clean reads were aligned to the reference genome using HISAT2 [36]. Gene expression levels were quantified using FeatureCounts [37] and normalized as FPKM values. RNA-seq data for *D. zingiberensis* were downloaded from the Genome Sequence Archive of the National Genomics Data Center (CRA007170) and analyzed as described above.

### 4.4 Phylogenetic analysis of NLRs

Amino acid sequences of the NB-ARC domain were extracted from the identified *Dioscorea* NLR proteins and aligned using MUSCLE [38]; poorly aligned regions were trimmed with TrimAl [39] using the -automated1 parameter. Phylogenetic analysis was performed with IQ-TREE [40] using the maximum likelihood method and the best-fit substitution model identified with ModelFinder. Branch support was assessed with 1000 ultrafast bootstrap replicates (-B 1000 --bnni).

### 4.5 Tracing gene duplication/loss events of NLR genes

The ancestral gene reconstruction was performed by reconciling the gene tree with the species tree of the three *Dioscorea* species, and NLR gene loss and duplication events were inferred using Notung (version 2.9.1.5) [25].

### 4.6 Distribution of NLR genes on chromosomes

The genomic locations of NLR genes on chromosomes were extracted from the GFF annotation files and visualized using the MapGene2Chroma webtool [41].

## CRediT authorship contribution statement

**Mengda Wang:** Writing – original draft, Methodology, Formal analysis, Data curation, Conceptualization. **Xiaowei Fu:** Validation, Methodology, Investigation, Formal analysis, Data curation, Conceptualization. **Li Yin:** Software, Resources. **Yi Zhao:** Software, Resources. **Qingjie Yang:** Investigation, Formal analysis. **Xuteng Ye:** Investigation, Formal analysis. **Jun Cheng:** Formal analysis, Data curation. **Daolong Dou:** Validation, Supervision, Funding acquisition. **Jinding Liu:** Writing – review & editing, Writing – original draft, Visualization, Investigation, Funding acquisition. **Gan Ai:** Writing – review & editing, Writing – original draft, Visualization, Validation, Supervision, Project administration, Methodology, Investigation, Funding acquisition, Formal analysis, Data curation, Conceptualization.

## Declaration of competing interest

The authors declare no competing interests.

## Data Availability

The RNA-seq and Ribo-seq data analyzed in this work were obtained from the NCBI Gene Expression Omnibus (https://www.ncbi.nlm.nih.gov/geo) under accession number GSE81332 and from the Genome Sequence Archive at the National Genomics Data Center, Beijing Institute of Genomics, Chinese Academy of Sciences, under accession number CRA007170 (https://bigd.big.ac.cn/gsa). NLRSeek can be obtained from GitHub (https://github.com/Wang-Mengda/NLRSeek).
