# Supplementary material for: NLRSeek: A reannotation–based pipeline for mining missing NLR genes in sequenced genomes
Source: aBIOTECH. 2025 Oct 17;7(1):100001. doi: 10.1016/j.abiote.2025.100001 (PMC12973398; doi:10.1016/j.abiote.2025.100001)
Supplement: Multimedia component 2 [file mmc2.docx]

**
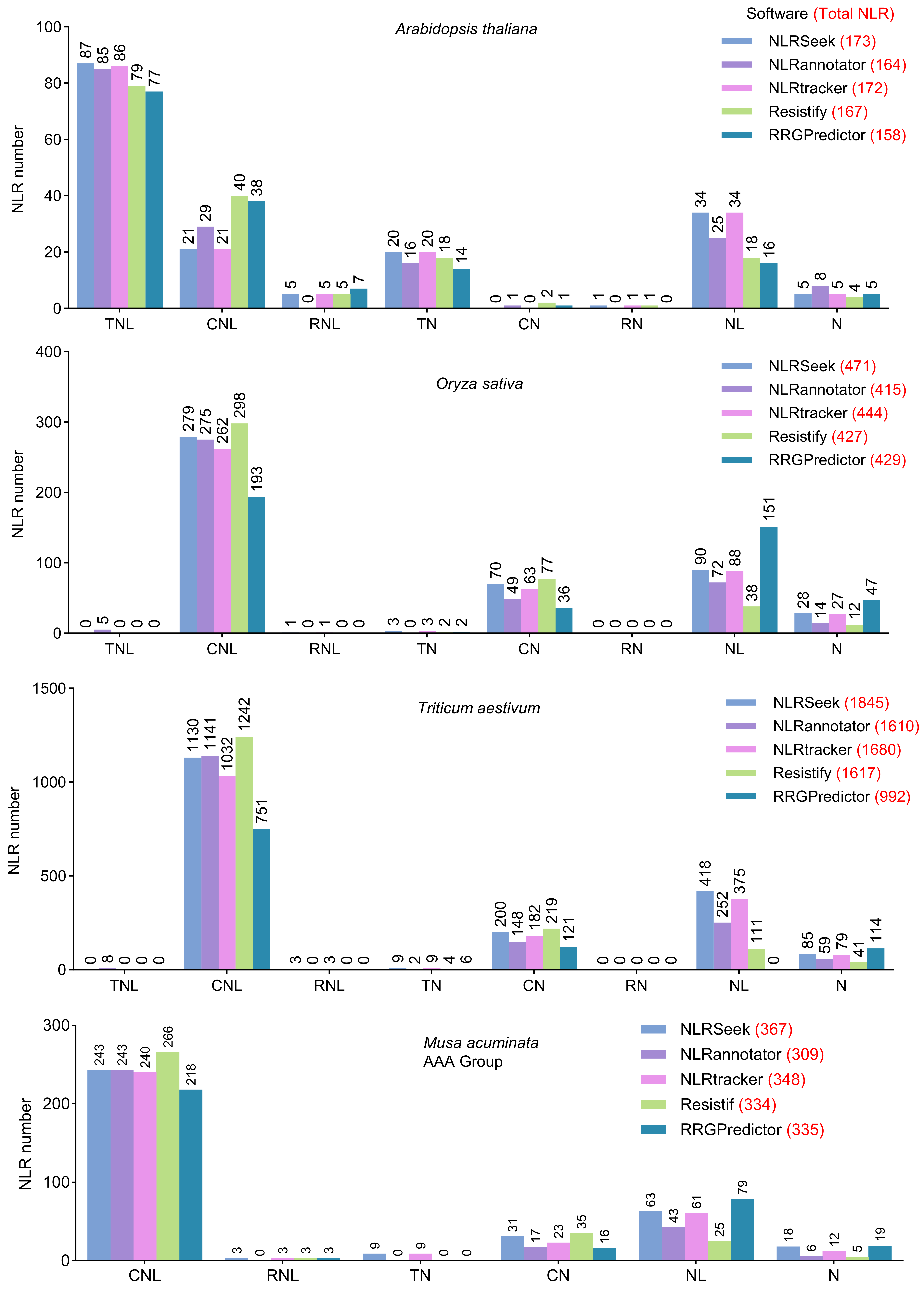
**

**Fig. S1** Comparison of NLRSeek with existing tools for NLR identification in Arabidopsis, rice, wheat and banana. CNL: CC-NB-ARC-LRR, RNL: RPW8-NB-ARC, TN: TIR-NB-ARC, CN: CC-NB-ARC, NL: NB-ARC-LRR, N: NB-ARC.


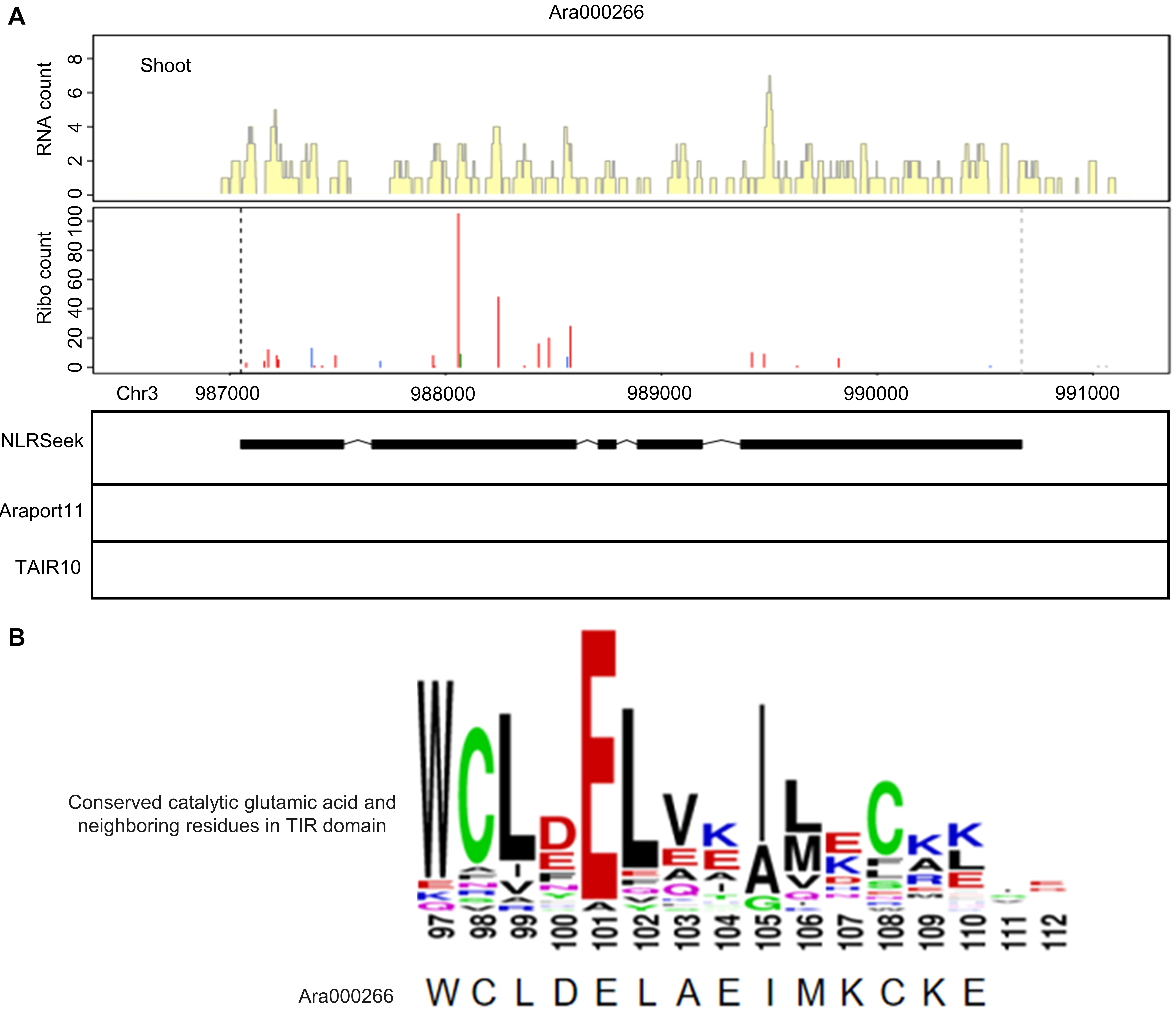


**Fig. S2** Ara000266 is a typical TNL.
**A** Expression and translation evidence for Ara000266. The genome annotations of Ara000266 locus from NLRSeek, Araport11, and TAIR10 are shown in the lower panel. RNA-seq and Ribo-seq data from Arabidopsis shoots were mapped to the genome. The upper panel displays genome browser views of RNA-seq and Ribo-seq signals. Y-axis indicates RNA-seq count and Ribo-seq P-site count. **B** Conservation of Ara000266 TIR domain. Weblogo of conserved catalytic glutamic acid and neighboring residues in TIR domain is shown. The residues in Ara000266 TIR domain are shown below.


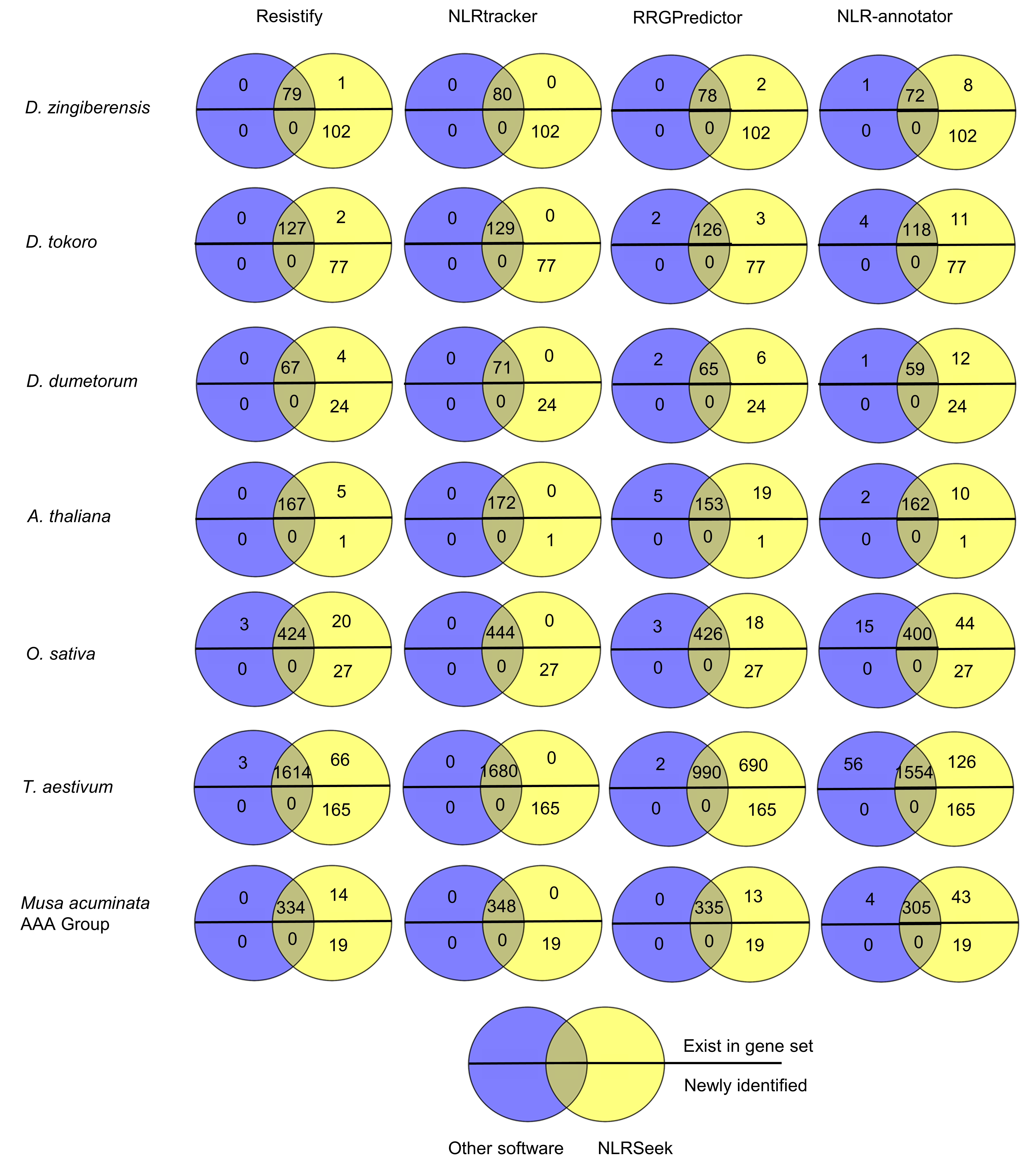


**Fig. S3** Overlap between NLRs identified by existing tools and NLRs identified by NLRSeek.

Species names are shown on the left. Venn diagrams illustrate the overlap between NLRs identified by existing tools and those identified by NLRSeek. The upper panels of venn diagrams show previously annotated genes in the gene sets, while the lower panels show newly annotated genes


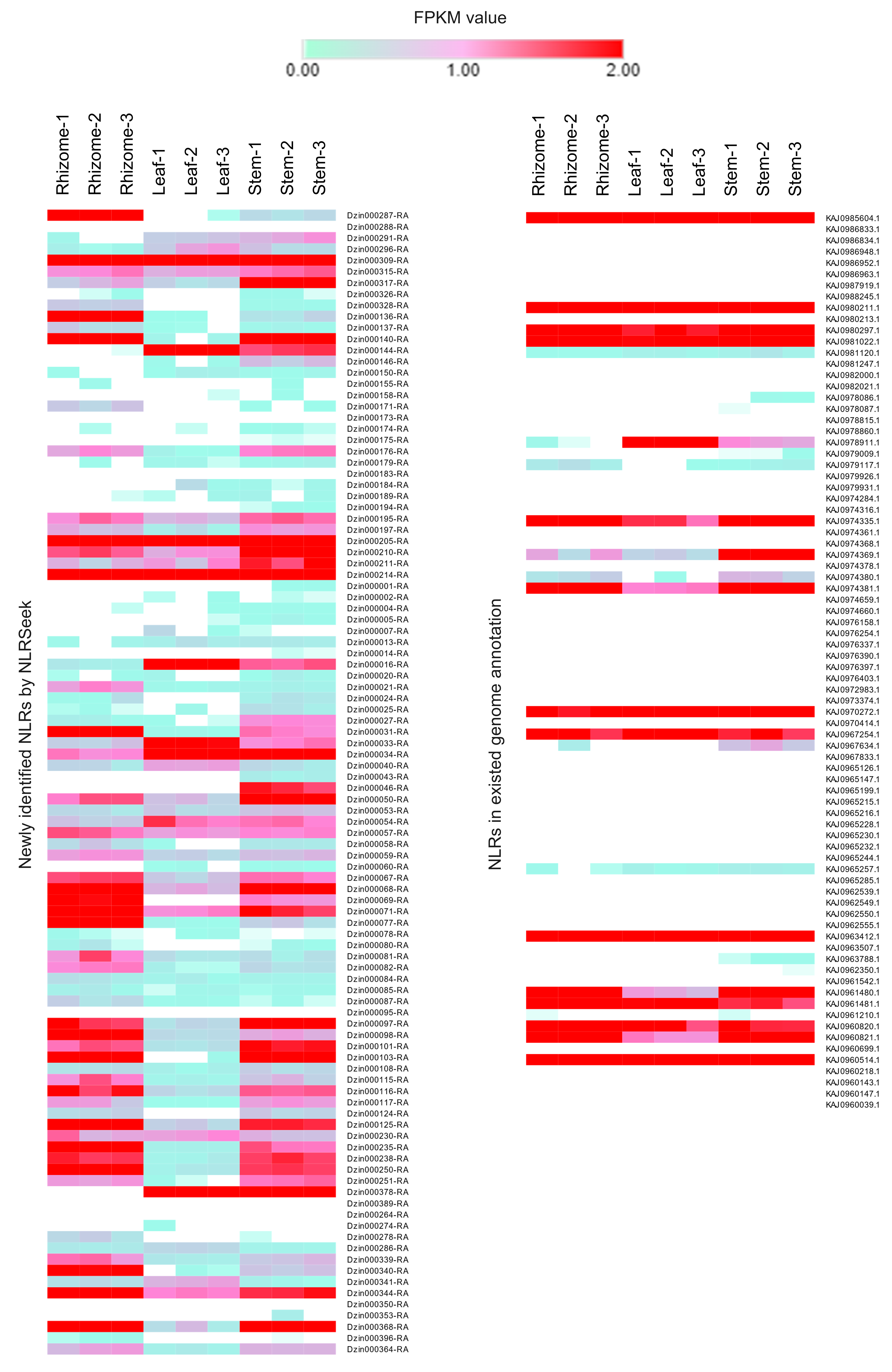


**Fig. S4** Previously mis-annotated NLRs in *D. zingiberensis* are actively transcribed genes.

Expression patterns of newly identified NLRs and NLRs from existing genome annotations are shown.

**
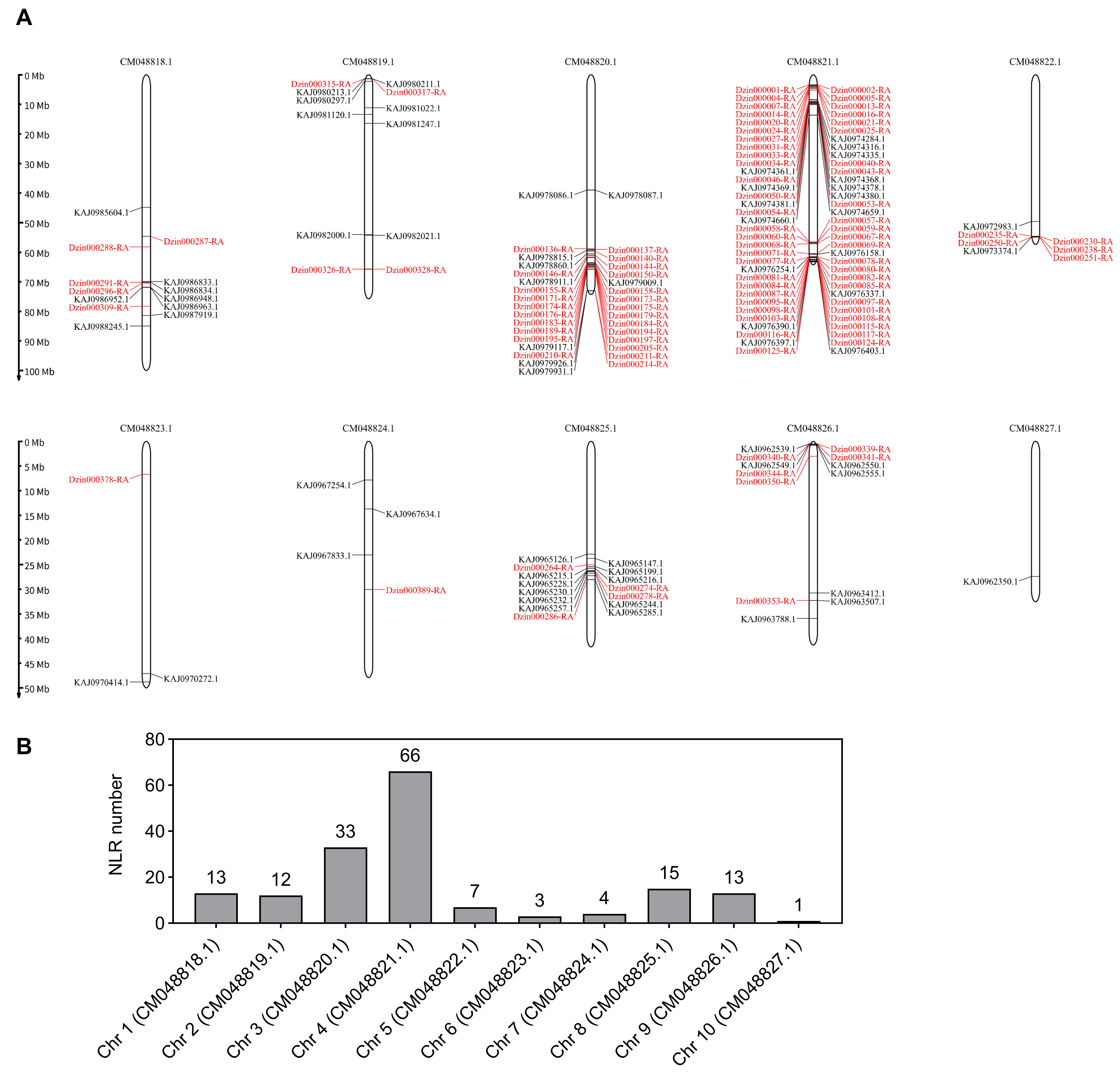
**

**Fig. S5** NLRs form clusters in the *D. zingiberensis* genome.

**A** Genomic distribution of NLRs in *D. zingiberensis*. Labels in red indicate newly annotated NLR genes identified by NLRSeek. **B** Number of NLR genes located on each chromosome.


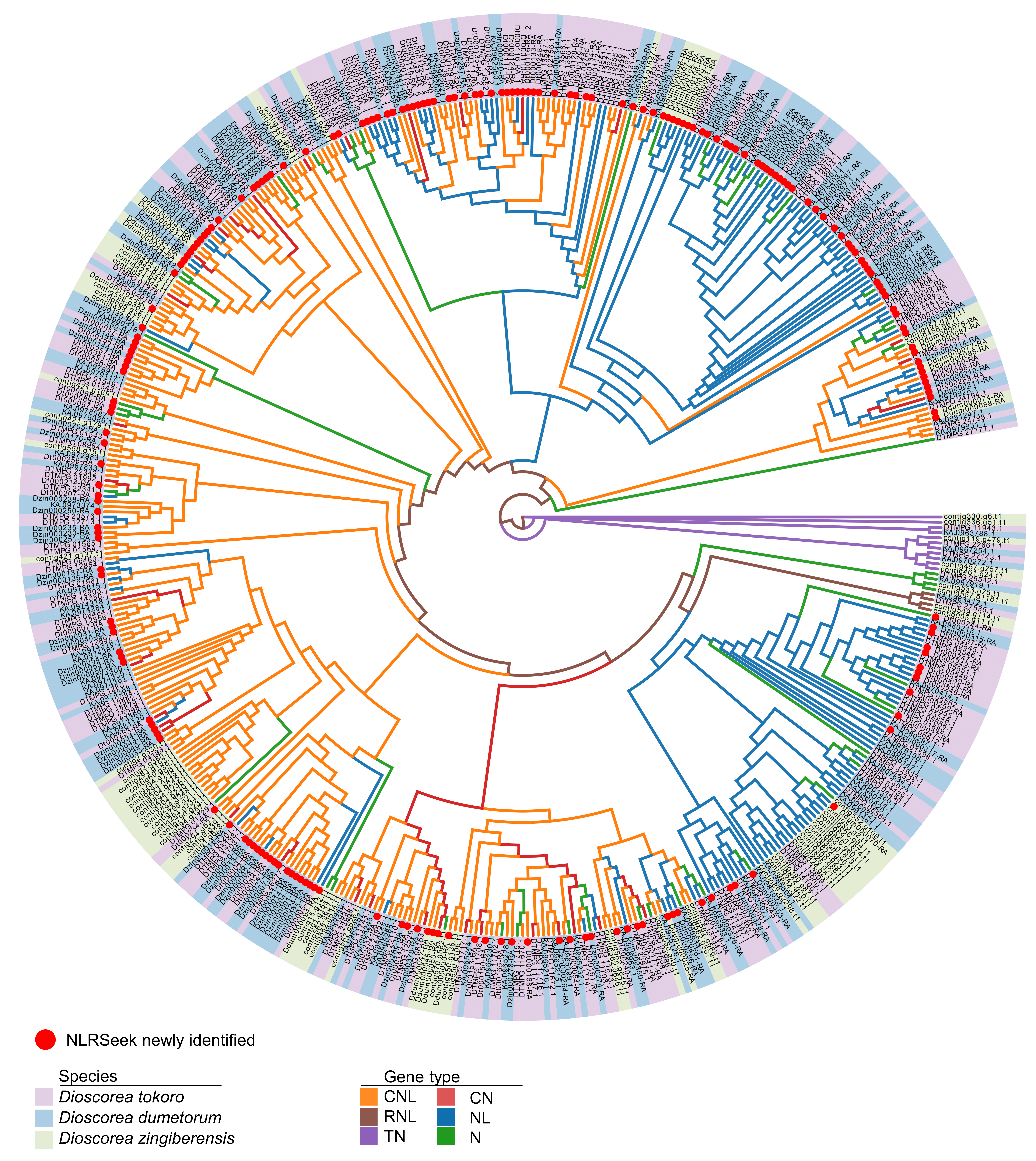


**Fig. S6** Phylogenetic tree of NLRs in three Dioscorea species.

NB-ARC domains were used to construct the tree. Multiple sequence alignment was performed using MUSCLE, and poorly aligned regions were trimmed with TrimAl using the -automated1 parameter. Phylogenetic analysis was conducted using IQ-TREE.
